# Metabolic outcomes in patients with type 2 diabetes mellitus after metabolic management center

**DOI:** 10.1097/MD.0000000000031342

**Published:** 2022-11-11

**Authors:** Xia Li, Xiaogang Bai, Jing Wang, Ting Bai, Yali Zhu, Sheli Li, Wenjing Ren, Xiaoling Niu, Jiayin Zheng, Changqin Kou

**Affiliations:** a Department of Endocrinology and Metabolism, Yan’an University Affiliated Hospital, Yan’an, Shaanxi, China.

**Keywords:** control rate, diabetes mellitus, glycosylated hemoglobin (HbA1c), national standardized metabolic management center (MMC)

## Abstract

The Chinese Medical Doctor Association has initiated metabolic management center (MMC) program for 6 years since 2016 nationwide. It is worth investigating the level of control metabolic outcomes in patients with type2 diabetes (T2DM) after MMC model in Yan’an, northwest China. Patients with T2DM was admitted to MMC in Yan’an University Affiliated Hospital from November 2018 to July 2021. They were asked to revisit hospital every 3 months. Blood glucose, blood pressure and blood lipids at baseline were compared to its counterparts after 1 year MMC management. Glycosylated hemoglobin and low density lipoprotein cholesterol (LDL-C) level in T2DM patients after 1 year management were lower than their baseline level (glycosylated hemoglobin 7.74 ± 1.94% vs 8.63 ± 2.26%, *P* < .001; LDL-C 1.81 ± 0.73mmol/L vs 2.18 ± 1.49mmol/L, *P* < .001). Mean HOMA-*β* increased after management (65.89 ± 90.81% vs 128.38 ± 293.93%, *P* < .05). After 1 year of management, patients in high school or above group achieved higher control rate of body mass index than those in middle school or below group (71.82% vs 28.18%, *P* = .043). high density lipoprotein cholesterol control rate was higher in high income group (42.86% vs 34.97%, 16.28%, *P* = .012), while LDL-C control rate was higher in low-income group (97.67% vs 78.57%, 84.51%, *P* = .018). fasting plasma glucose control rate in new diagnosis group was higher than that of the middle and long course groups (71.43% vs 52.38%, 42.44%, *P* = .002). The comprehensive control rate increased from 9.83% at baseline to 26.15% after 1 year MMC management. The metabolic outcomes and their control rate in T2DM patients were improved after 1 year MMC management. It indicated that patients may achieve more benefits with MMC management.

## 1. Introduction

With the rapid development of China’s economy and the westernization of people’s lifestyle, the prevalence of type 2 diabetes mellitus (T2DM) has risen to 11.2% in 2017, but the overall control rate of diabetes is still at a very low level.^[1]^ Individually, patients with T2DM failed to adopt a healthy lifestyle and stick to regular treatment may account for it.^[2]^ In addition, the disparity in level of T2DM diagnosis and treatment in different areas of China attributed to it.^[3]^

In order to achieve the homogenization management of diabetes and raise the control rate of diabetes, Chinese Medical Doctor Association launched the Metabolic Management Center (MMC) program in 2016, which combined advanced diagnosis and treatment technology with big data sharing patients’ information online and offline among different hospitals. It is a new mode for T2DM management adapted to China’s grading treatment.^[4]^ Studies have shown that MMC management could improve metabolic level for both outpatients and in-patients with (T2DM) in China’s developed areas, especially for blood glucose and glycosylated hemoglobin (HbA1c).^[5–7]^

Yan’an University Affiliated Hospital, located in Northwest China, is the largest medical service center in Northern Shaanxi, a less developed area. It joined MMC in November 2018 and was officially awarded the MMC sub-center in August 2019. The purpose of the study is to analyze the prognosis of T2DM patients after MMC management, and further to understand the current management status of T2DM patients in less developed areas of Northwest China.

## 2. Method

### 2.1. Patient recruitment

Patients with T2DM who were managed in sub-MMC center from November 2018 to July 2021 were recruited in the study. Patients were included as the following criteria: Patients aged 18 to 70 who were diagnosed with type 2 diabetes according to the World Health Organization criteria in 1999.^[8]^ Accepted and adhered to MMC management for more than 1 year. Was able to download and use MMC app. Patients with the following conditions were excluded from the study: diabetic ketoacidosis, severe infection, acute cardiovascular and cerebrovascular diseases, acute pancreatitis, Type 1 diabetes mellitus, gestational diabetes mellitus. The study was approved by the Ethics Committee of Yan’an University Affiliated Hospital and was conducted in accordance with the principles of the Declaration of Helsinki.

### 2.2. MMC management

Patients who meet the criteria above were guided to download and use the MMC app for appointments, follow-up and self-management. Their demographic information was collected and physical measurements recorded by MMC specified nurses. Their clinical examinations, diagnosis and treatment plan were completed by doctors. Patients went through a 60-minute diabetes education in MMC. Patients are requested to visit the hospital every 3 months for physical measurements, clinical examinations and diabetic education. After 1 year, all the patients returned to the hospital for health examination and data collection.

### 2.3. Baseline characteristics

Gender, date of birth, current address, education level, family income and other demographic information were collected when patients joined MMC. Physical measurements including height, weight, waist circumference, systolic blood pressure (SBP), diastolic blood pressure (DBP), and body mass index (BMI) were recorded when patients joined and revisited MMC.

We categorized the patients based on their demographic information in this study education level was divided into 2 groups, junior middle school or less and senior high school or above. Family income levels were divided into low-income group (10,000–30,000 RMB/year), middle-income group (30,000–1,00,000 RMB/year) and high-income group (>1,00,000 RMB/year). Duration of diabetes was divided into < 1 year, 1 to 5 years and > 5 years. In this study, duration of diabetes < 1 year was also defined as a new diagnosis, 1 to 5 years was defined as a medium course of T2DM, and > 5 years was defined as a long course of T2DM.

### 2.4. Laboratory examination

Venous blood was collected after at least 8 hours’ fasting. Fasting blood glucose (FBG), 2-hour postprandial blood glucose (2hPG),HbA1c, total cholesterol (TC), triglycerides (TG), high density lipoprotein cholesterol (HDL-C), low density lipoprotein cholesterol (LDL-C) were tested in the central laboratory of the hospital. Plasma glucose level was determined by glucose oxidase method. Serum insulin level was examined by radioimmunoassay. Blood lipid profile was measured by standard enzymatic test.

We conducted insulin release test among those whose blood glucose have been controlled. Blood glucose, insulin and C-peptide were examined for fasting level and postprandial level after breakfast. Oral glucose tolerance test was performed on those who need to make a definite diagnosis. The improved steady-state model [omeostis model assessment for insulin resistance (C-peptide), HOMA-IR (CP)] was adopted to calculate the insulin resistance index and the formula as following: HOMA-IR (CP) = 1.5 + fasting blood glucose × fasting C peptide/2800. The modified steady-state model was used to calculate islets β cell function index [homeostasis model assessment for β-cell function (C-peptide), HOMA-β (CP)] and its formula HOMA-β (CP) = 0.27 × fasting C-peptide/ (fasting blood glucose – 3.5).^[9]^

According to the guideline for prevention and treatment of type 2 diabetes in China by Chinese Diabetes Society, patients with T2DM were considered to be controlled as the following: fasting plasma glucose (FPG) 4.4 to 7.0 mmol/L, non fasting plasma glucose < 10.0mmol/L, HbA1c < 7.0%; SBP < 130 mm Hg, DBP < 80 mm Hg; TC < 4.5 mmol/L, TG < 1.7mmol/L; HDL-C > 1.0mmol/L for male, 1.3 mmol/L for female; LDL-C < 2.6 mmol/L for patients without atherosclerotic cardiovascular disease and 1.8 mmol/L for patients with atherosclerotic cardiovascular disease; BMI < 24.0 kg/m^2^.^[10]^

### 2.5. Statistical analysis

In the study, we compared physical measurement and laboratory tests among patients before and after MMC management for 1 year. Continuous data distributed normally were described as mean ± standard deviation, tested by *t*-test for 2 groups and analysis of variance for 3 or more groups. Continuous data distributed abnormally were described as median (interquartile interval) and tested by Wilcoxon test for 2 groups and Kruskal–Wallis test for 3 groups. Categorical variables are presented as frequency and percentages and tested by *χ*^2^ test. *P* < .05 was considered statistically significant in the study.

## 3. Results

### 3.1. Baseline of the patients

A total of 363 patients with T2DM were included in the study, in which 276 (76.03%) were males and 87 (24.97%) were females. More than 80% of male patients had high school or above education level. The average level of HbA1c and HOMA-IR in patients with high school or above was lower than those in patients with middle school or less. More male patients were in high-income group (83.84%). The average level of HbA1c, HOMA-IR, HOMA-β in high-income group was lower than that in low-income group. The average SBP of patients with long course of T2DM was higher than that of moderate course and newly diagnosis group (129.06 + 17.14 mm Hg, 123.78 + 14.31 mm Hg, 122.93 + 16.06 mm Hg). FPG (10.21 ± 14.31mmo/L), HbA1c (9.51 ± 2.61%), TC (4.76 ± 1.06mmol/L) and LDL-C (2.82 ± 2.62mmol/L) in the newly diagnosed patients were higher than those in the other 2 groups. Table 1.

**Table 1:** Baseline characteristics of patients before MMC management (Mean ± SD, n).

Groups	Sex male/female			Age	Body weight		BMI		Waist circumference	
Education level										
Junior middle school or less	90/42			52.19 ± 12.15	69.99 ± 12.37		25.42 ± 5.44		90.48 ± 8.66	
Senior high school or above	173/41			52.64 ± 11.62	70.69 ± 11.76		24.55 ± 3.40		90.60 ± 8.98	
* P*	.007			.733	.601		.069		.901	
Duration of diabetes										
<1 yr	52/17			46.40 ± 14.75	71.19 ± 12.54		25.05 ± 3.54		90.20 ± 9.55	
1–5 yr	70/21			49.41 ± 11.22	72.74 ± 14.73		25.60 ± 5.53		91.77 ± 10.21	
>5 yr	154/49			56.34 ± 10.53	69.13 ± 10.29		24.48 ± 3.78		90.20 ± 8.08	
*P*	.971			<.001	.063		.137		.589	
Family income										
Low-income	39/25			55.92 ± 9.68	69.86 ± 10.87		24.72 ± 3.57		89.98 ± 7.63	
Middle-income	140/41			53.98 ± 13.02	69.78 ± 12.53		24.83 ± 4.41		90.66 ± 9.14	
High-income	83/16			53.90 ± 10.78	71.84 ± 11.67		25.03 ± 4.62		90.71 ± 9.17	
*P*	.003			.537	.361		.892		.851	

Groups	SBP		DBP			FPG (mmol/L)		2hPG (mmol/L)		HbA1c (%)
Education level										
Junior middle school or less	126.75 ± 17.16		77.43 ± 9.76			7.94 ± 2.74		14.09 ± 4.13		8.93 ± 2.41
Senior high school or above	126.64 ± 16.04		77.93 ± 10.89			8.27 ± 8.39		14.33 ± 8.50		8.37 ± 2.07
*P*	.954		.665			.671		.781		.024
Duration of diabetes										
<1 yr	122.93 ± 16.06		77.26 ± 9.49			10.21 ± 14.31		15.80 ± 13.90		9.51 ± 2.61
1–5 yr	123.78 ± 14.31		77.85 ± 10.05			7.63 ± 2.45		12.78 ± 3.54		8.13 ± 2.27
>5 yr	129.06 ± 17.14		77.70 ± 11.04			7.67 ± 2.37		14.31 ± 3.80		8.49 ± 1.96
*P*	.006		.870			.045		.112		.005
Family income										
Low-income	125.42 ± 17.17		75.22 ± 13.53			7.87 ± 2.58		14.30 ± 3.31		8.95 ± 2.26
Middle-income	127.15 ± 16.79		78.30 ± 9.79			7.92 ± 3.19		14.80 ± 9.43		8.72 ± 2.32
High-income	126.26 ± 15.37		78.36 ± 9.29			8.73 ± 11.73		13.29 ± 3.62		8.14 ± 1.93
*P*	.751		.102			.612		.300		.045

Groups	TC	TG	HDL			LDL		HOMA-IR		HOMA-β
Education level										
Junior middle school or less	4.47 ± 1.11	2.49 ± 6.01	1.16 ± 4.21			3.27 ± 30.12		5.07 ± 9.07		78.84 ± 160.18
Senior high school or above	4.43 ± 1.22	3.26 ± 16.43	0.98 ± 0.32			2.08 ± 1.09		3.29 ± 3.95		59.47 ± 70.84
*P*	.588	.103	.205			.427		.022		.158
Duration of diabetes										
<1 yr	4.76 ± 1.06	2.04 ± 1.14	1.01 ± 0.34			2.82 ± 2.62		4.52 ± 8.18		72.61 ± 182.54
1–5 yr	4.51 ± 1.18	3.85 ± 15.17	1.01 ± 0.29			2.24 ± 0.87		2.92 ± 2.32		52.98 ± 53.23
>5 yr	4.23 ± 1.15	2.02 ± 1.84	1.59 ± 7.53			2.02 ± 0.79		4.08 ± 6.68		91.85 ± 105.20
*P*	.007	.201	.634			.001		.422		.018
Family income										
Low-income	4.35 ± 1.02	2.10 ± 1.43	0.96 ± 0.25			2.03 ± 0.73		5.96 ± 10.36		106.55 ± 223.22
Middle-income	4.35 ± 1.14	2.87 ± 10.95	1.06 ± 0.33			2.30 ± 1.79		3.71 ± 5.59		58.57 ± 69.11
High-income	4.53 ± 1.23	2.17 ± 2.00	2.10 ± 10.71			2.17 ± 0.80		3.19 ± 4.04		56.85 ± 59.81
*P*	.467	.703	.310			.406		.035		.018

BMI = body mass index, DBP = diastolic blood pressure, FPG = fasting plasma glucose, 2hPG = 2-hour postprandial blood glucose, HbA1c = glycosylated hemoglobin, HOMA = homeostasis model assessment, HDL = high density lipoprotein cholesterol, LDL = low density lipoprotein cholesterol, MMC = metabolic management center, SD = standard deviation, SBP = systolic blood pressure, TC = total cholesterol, TG = triglycerides.

### 3.2. Metabolic outcomes changes

The average HbA1c was 8.63 ± 2.26% versus 7.74 ± 1.94% (*P* < .001) in patients before and after MMC management for 1 year, LDL-C was 2.18 ± 1.49 mmol/L versus 1.81 ± 0.73 mmol/L (*P* < .001), Waist circumference was 90.28 ± 9.04cm versus 88.65 ± 9.37cm (P < .05), HOMA-β was 65.89 ± 90.81% versus 128.38 ± 293.93% (*P* < .05). However, body weight, BMI, FPG, 2hPG, DBP, TC, TG and HDL-C showed no significant changes before and after the management (*P* > .05) (Table 2).

**Table 2:** Metabolic outcomes before and after 1 year of MMC management (mean ± SD).

Indicators	Before MMC management	After MMC management	*t*	*P*
Body weight (kg)	70.50 ± 12.48	70.22 ± 13.18	0.305	.761
BMI (kg/m^2^)	24.93 ± 4.58	24.44 ± 3.06	1.670	.096
Waist circumference (cm)	90.28 ± 9.04	88.65 ± 9.37	2.479	.014
SBP (mm Hg)	126.53 ± 16.54	129.16 ± 16.39	2.050	.041
DBP (mm Hg)	77.38 ± 10.67	78.90 ± 10.24	1.794	.074
FPG (mmol/L)	8.29 ± 8.04	8.45 ± 9.70	0.179	.858
2hPG (mmol/L)	14.49 ± 9.01	13.88 ± 3.95	0.795	.428
HbA1c (%)	8.63 ± 2.26	7.74 ± 1.94	6.276	<.001
TG (mmol/L)	2.54 ± 8.79	2.07 ± 2.64	0.856	.393
TC (mmol/L)	4.34 ± 1.13	4.29 ± 1.21	0.560	.576
LDL-C (mmol/L)	2.18 ± 1.49	1.81 ± 0.73	3.889	<.001
HDL-C (mmol/L)	1.43 ± 6.42	1.00 ± 0.31	1.119	.264
HOMA-IR	3.74 ± 6.38	6.13 ± 10.13	2.596	.010
HOMA-β	65.89 ± 90.81	128.38 ± 293.93	2.513	.013

BMI = body mass index, DBP = diastolic blood pressure, FPG = fasting plasma glucose, 2hPG = 2-hour postprandial blood glucose, HbA1c = glycosylated hemoglobin, HDL-C = high density lipoprotein cholesterol, HDL = high density lipoprotein cholesterol, HOMA = homeostasis model assessment, LDL = low density lipoprotein cholesterol, LDL-C = low density lipoprotein cholesterol, MMC = metabolic management center, SBP = systolic blood pressure, TG = triglycerides, TC = total cholesterol.

### 3.3. Control rate in different demographics

The control rate of BMI in patients with high school or above was higher than that in middle school or less group (71.82% vs 28.18%, *χ*^2^ = 4.102, *P* = .043). The control rate of HDL-C in patients with high, medium and low family income were 42.86%, 34.97% and 16.28% (*χ*^2^ = 8.934, *P* = .012), while the control rate of LDL-C were 78.57%, 84.51% and 97.67% (*χ*^2^ = 8.049, *P* = .018), respectively. Compared with patients with medium or long course of T2DM, the control rate of FPG in patients with newly diagnosis was the highest (71.43%, *χ*^2^ = 5.996, *P* = .002). Table 3.

**Table 3:** Control rate of metabolic outcomes after 1 year of MMC management (n, %).

Groups	BMI	Blood pressure	FPG	HbA1C	TC	TG	HDL-C	LDL-C
Education level								
Junior middle school or less	31 (28.18)	77 (58.33)	42 (50.00)	56 (49.12)	67 (66.34)	59 (58.42)	29 (28.71)	88 (87.13)
Senior high school or above	79 (71.82)	110 (51.40)	72 (50.70)	92 (46.23)	96 (56.47)	106 (61.99)	64 (37.65)	141 (83.43)
*P*	.043	.209	.919	.622	.109	.560	.134	.413
Duration of diabetes								
<1 yr	22 (37.93)	33 (45.21)	35 (71.43)	39 (57.35)	35 (61.40)	34 (58.62)	22 (38.60)	46 (80.70)
1–5 yr	20 (45.55)	34 (51.52)	22 (52.38)	27 (48.21)	26 (54.17)	30 (62.50)	14 (29.17)	39 (82.98)
>5 yr	68 (42.77)	121 (58.17)	57 (42.22)	83 (43.46)	103 (61.68)	102 (61.08)	57 (34.13)	145 (86.83)
*P*	.808	.143	.002	.142	.633	.914	.597	.497
Family income								
Low-income	20 (45.45)	21 (47.73)	20 (51.28)	24 (43.64)	27 (62.79)	24 (55.81)	7 (16.28)	42 (97.67)
Middle-income	49 (35.51)	51 (36.96)	60 (50.85)	77 (48.13)	89 (62.24)	88 (61.11)	50 (34.97)	120 (84.51)
High-income	40 (49.38)	33 (40.74)	34 (50.00)	46 (47.92)	47 (55.95)	52 (61.90)	36 (42.86)	66 (78.57)
*P*	.111	.439	.990	.838	.607	.784	.012	.018

BMI = body mass index, FPG = fasting plasma glucose, HbA1c = glycosylated hemoglobin, HDL = high density lipoprotein cholesterol, LDL = low density lipoprotein cholesterol, LDL-C = low density lipoprotein cholesterol, MMC = metabolic management center, TC = total cholesterol, TG = triglycerides.

### 3.4. Control rate before and after MMC management

**Figure 1.**
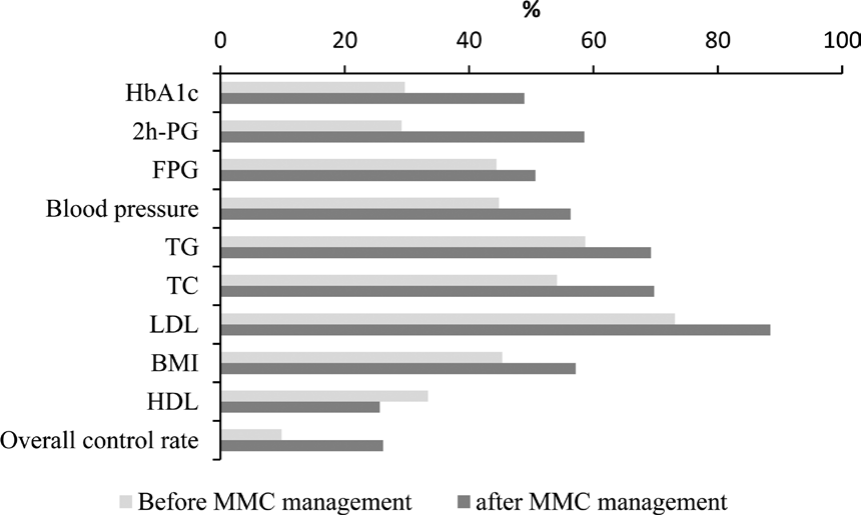
Control rates of metabolic outcomes before and after MMC management. MMC = metabolic management center.

The control rate of HbA1c in patients was 29.67% versus 48.90% (*χ*^2^ = 28.219, *P* < .001) before and after 1 year of management, the rate of blood pressure was 44.78% versus 56.32% (*χ*^2^ = 9.694, *P* = .002), TG was 58.67% versus 69.23% (*χ*^2^ = 11.940, *P* = .001), TC was 54.12% versus 69.78% (*χ*^2^ = 18.933, *P* < .001), LDL-C was 73.08% versus 88.46% (*χ*^2^ = 27.733, *P* < .001), BMI was 45.33% versus 57.14% (*χ*^2^ = 10.166, *P* = .001). The overall control rate increased from 9.83% before management to 26.15% after management (*χ*^2^ = 31.303, *P* < .0019) (Table 4, Fig. 1).

**Table 4:** Control rates of metabolic outcomes before and after MMC management (n, %).

Indicators	Before MMC management n (%)	After MMC management n (%)	*χ^2^*	*P*
FPG	151 (44.41)	115 (50.66)	2.134	.144
2h-PG	106 (29.12)	213 (58.52)	63.883	<.001
HbA1c	108 (29.67)	178 (48.90)	28.219	<.001
Blood pressure	163 (44.78)	205 (56.32)	9.694	.002
TG	207 (58.67)	252 (69.23)	11.940	.001
TC	197 (54.12)	254 (69.78)	18.933	<.001
HDL-C	143 (33.39)	93 (25.62)	15.695	<.001
LDL-C	266 (73.08)	322 (88.46)	27.733	<.001
BMI	165 (45.33)	208 (57.14)	10.166	.001
Overall control rate	34 (9.83)	91 (26.15)	31.303	<.001

BMI = body mass index, FPG = fasting plasma glucose, 2hPG = 2-hour postprandial blood glucose, HbA1c = glycosylated hemoglobin, HDL = high density lipoprotein cholesterol, LDL = low density lipoprotein cholesterol, LDL-C = low density lipoprotein cholesterol, MMC = metabolic management center, TG = triglycerides, TC = total cholesterol.

## 4. Discussion

MMC is a new mode for T2DM management which adopted information platform and new media to the program. It could carry out the diagnosis and long-term follow-up for T2DM patients in a new 1-stop management model. After 1 year of MMC management, the level of blood glucose and blood lipids of the patients improved, HbA1c and LDL-C were significantly lower than before management. And HOMA-β increased significantly. The control rate of multiple metabolic outcomes in T2DM was also improved in the study.

Metabolic parameters were improved after the MMC program, which require patients’ involvement to the treatment process. Similarly, better disease control achievement were possible by training and management of the patients in T2DM and hypertension.^[11,12]^ The decreased HbA1c after management were consistent with HbA1c change after MMC follow-up management in MMC sub-centers in Ningbo Affiliated First hospital.^[7]^ FPG, 2hPG and other metabolic outcomes showed no changes before and after management, especially 2hPG, which might be related to the fact that all 2hPG test in the study were 2-hour blood glucose by steamed bread meal insulin release test. No hypoglycemic drugs used during the test resulted in overall high postprandial blood glucose. Moreover, insignificantly elevated HDL cholesterol was reported in our study after implementation of MMC. Similarly, a study showed that initiation of Sodium glucose co-transporter-2 inhibitor treatment also insignificantly improves HDL levels. In contrast, significant lower levels of HDL have been reported in patients with T2DM.^[13]^ Significant reduction in LDL has been noted in present study, similar to noted in previous reports.^[14]^ Most patients with T2DM in the present study have atherosclerosis and were treated with oral statins for lipid regulation and plaque stabilization, resulting LDL-C decreased. In general, the level of HbA1c, blood pressure, TG, TC, LDL-C and BMI in patients with T2DM after MMC management were lower than those before MMC management, and HbA1c control rate increased from 29.67% to 48.90%. The comprehensive control rate increased from 9.83% to 26.15%. It is proved that MMC management could improve the condition of diabetes patients.

Control of blood glucose is critical for T2DM management. Results in the study showed the control rate improved overall after MMC management. HbA1c is one of the main metabolic indicators reflecting long-term glycemic control. The average HbA1c for majority of the patients with T2DM aimed to be less than 7%.^[15]^ Blood pressure, blood lipid and weight management for patients with T2DM should be individualized and be considered comprehensively in treatment plan.^[1]^ A study showed that from 2013 to 2016, 64% of T2DM patients achieved HbA1c control, 70% achieved blood pressure control and 57% achieved LDL-C respectively, but only 23% T2DM patients achieved the comprehensive control, similarly to the rate in our study.^[16]^ Evidence on international diabetes management practice suggested that the number of HbA1c less than 7% decreased from 36% in 2005 to 30.1% in 2017 among T2DM patients from 49 developing countries, indicating that the level of blood glucose control in developing countries has been worsen in the past 12 years.^[17]^ Another study evaluated 1512 T2DM patients in China showed that the control rates of FPG, 2hPG and HbA1c were 25.5%, 22.7% and 19.5%, respectively. And the overall control rate of DM patients was still low.^[18]^ Similarly, low control rates among type 2 DM patients were found in other studies.^[19,20]^ However, a study in MMC center indicated that the average life expectancy in the management group in MMC would be 0.61 years higher than that of the control group in the next 30 years through UKPDS (The United Kingdom Prospective Diabetes Study) model simulation.^[5]^ Our study added evidence that MMC program could improve the control rate of HbA1c and other metabolic outcomes in patients with T2DM. We proposed that clinical modes, especially for non-chronic disease, providing knowledge and skills with strict follow-ups for patients could enhance clinical outcomes. The modes may adopt information platform or new media.

There was no significant difference in HbA1c, waist circumference, BMI, blood pressure, TC, TG and other indexes of T2DM patients with different demographics after MMC management, indicating that demographic characteristics might have no significant impact on metabolic outcomes of the patients. The findings were accorded with the results from the National Health and Nutrition Examination Survey (NHANES) from 1999 to 2010.^[21]^ However, compared with the middle and long course of duration groups, the FPG control rate of newly diagnosed T2DM patients was higher. The newly diagnosed patients might be younger, have better recovery of their islet *β* cell function after treatment. It was consistent with the results of MMC in Shanghai No.10 Hospital.^[22]^ Besides FPG, the study found that other indicators have no obvious differences among different demographics. On one hand, the management of MMC, including receiving the same diabetic education, standardization of diagnosis and treatment, and regular follow-up, might improve the overall conditions of the patients. On the other hand, it may also because the study ruled out patients with severe acute, chronic diabetic complications and patients with life expectancy < 5 years.

This study has some limitations. The study only had patients who joined MMC program and compared their treatment effects before and after MMC. A control group of patients from conventional management mode should be involved in the future study. All the patients were those who visited the hospital for diabetic treatment. Sample size calculation omitted from the study. The patients in the study were often male and with high income, and this might have caused a bias in selection.

The management of MMC emphasized providing knowledge and skills for patients with diabetes and has strict follow-up plans and procedures. Health education and close doctor-patient relationship greatly improved the compliance of patients with diabetes, which is conducive to delaying complications and reducing medical costs. Based on our study, we found MMC management benefited patients with T2DM in this area. It is necessary to continue to assess the treatment and control effect and expand this management model to other parts of China.

## Author contributions

**Conceptualization:** Xiaogang Bai, Jing Wang, Ting Bai, Yali Zhu, Sheli Li.

**Data curation:** Yali Zhu.

**Formal analysis:** Xia Li.

**Investigation:** Jing Wang, Ting Bai, Sheli Li.

**Methodology:** Xia Li, Ting Bai, Sheli Li, Xiaoling Niu.

**Project administration:** Xiaogang Bai, Jing Wang, Yali Zhu.

**Resources:** Wenjing Ren, Xiaoling Niu, Jiayin Zheng, Changqin Kou.

**Supervision:** Sheli Li, Wenjing Ren, Xiaoling Niu, Jiayin Zheng.

**Software:** Wenjing Ren.

**Validation:** Wenjing Ren, Xiaoling Niu, Changqin Kou.

**Writing – original draft:** Xia Li.

**Writing – review & editing:** Xia Li, Xiaoling Niu, Jiayin Zheng, Changqin Kou.
